# Emergence and Rapid Spread of Tetracycline-Resistant *Vibrio cholerae* Strains, Madagascar

**DOI:** 10.3201/eid0803.010258

**Published:** 2002-03

**Authors:** Jacques-Albert Dromigny, Olivat Rakoto-Alson, Davidra Rajaonatahina, René Migliani, Justin Ranjalahy, Philippe Mauclère

**Affiliations:** * Institut Pasteur de Madagascar, Antananarivo, Republic of Madagascar; and †Ministry of Health, Antananarivo, Republic of Madagascar

**To the Editor:** The Indian Ocean was free of cholera for decades, until January 1998, when an outbreak was detected in Comoros Islands (*1*). On March 23, 1999, the Malagasy Epidemiological Surveillance System reported the first case of cholera in Mahajanga, a harbor on the northwest coast (*2*). In May 1999, the Malagasy sanitary authorities set up sanitary barricades at the borders of the two provinces—Mahajanga and Antananarivo—affected by the epidemic. Oral doxycycline was systematically given to all the travelers crossing the barricades. In addition, doctors in hospitals and dispensaries in these two provinces gave doxycycline to patients with acute diarrhea. Despite these measures, cholera had reached all six provinces of the island 10 months later. In June 1999, a specific cholera surveillance system was established in every Malagasy province with close collaboration between the Malagasy Ministry of Health and the Institut Pasteur de Madagascar.

The first strain isolated in Mahajanga was *Vibrio cholerae* serogroup O1, serotype Ogawa, biotype El Tor. Its antibiotype showed resistance to trimethoprim-sulfamethoxazole, sulfonamides, trimethoprim, chloramphenicol, streptomycin, and vibriostatic agent O129 (a molecule naturally active against *V. cholerae* and used for identification). Susceptibility was conserved for tetracycline, ampicillin, cephalotin, and pefloxacin (*2*). This strain showed a rRNA gene restriction pattern similar to those of African and Comorian strains isolated since 1994 and 1998, respectively (*2*,*3*).

From July 1999 to March 2001, we monitored the tetracycline resistance of *V. cholerae* isolated from the stool samples sent to the Institut Pasteur de Madagascar in Antananarivo, using the standard disk-diffusion method (*4*). Stool samples were collected in sterile containers, on Whatman paper, or on rectal swabs. Isolation of *V. cholerae* was carried out immediately after reception. Every *V. cholerae* strain identified belonged to serogroup O1, biotype El Tor. All the tetracycline-resistant *V. cholerae* isolated and 60 randomly selected tetracycline-susceptible strains were tested for sensitivity to the following drugs : ampicillin, cephalotin, doxycycline, sulfonamide, trimethoprim, trimethoprim-sulfamethoxazole, chloramphenicol, streptomycin, spectinomycin, neomycin, kanamycin, nalidixic acid, pefloxacin, erythromycin, rifampicin, and nitrofurantoin, as well as to vibriostatic agent 0129.

During the study period, we isolated 351 (46.1%) *V. cholerae* strains from 761 stool samples analyzed. The provinces of Antananarivo, Mahajanga, and Toliary accounted for 85.9% of the stool samples sent to our laboratory. From these provinces, we isolated 288 strains; by contrast, from the three other provinces (Antsiranana, Fianarantsoa, and Toamasina, located on the east coast), 63 strains were isolated. Rates of isolation, tested by a chi-square test, did not differ significantly between the six provinces (p=0.32).

Fifty five (15.7%) of the 351 strains isolated were found to be tetracycline resistant (cross-resistance with doxycycline) but had the same resistance pattern as the index strain isolated in Mahajanga for the other antibiotics tested. During the first rainy season following the epidemic (November 1999 to March 2000), a unique tetracycline-resistant strain was isolated (in February 2000), in the capital Antananarivo; it was also resistant to ampicillin, nalidixic acid, and nitrofurantoin. During the dry season (from April to October 2000), five (13.2%) of 38 *V. cholerae* new tetracycline-resistant strains were found. However, during the last rainy season (November 2000 to March 2001), 49 (69 %) of 71 strains isolated were tetracycline resistant. They were mainly from the city and suburbs of Antananarivo (95.3%, 41/43 strains). The eight other resistant strains came from the provinces of Antananarivo, Toliary, and Fianarantsoa.

As observed in Tanzania (*5*), the extensive prophylactic use of tetracycline may have triggered the rapid emergence and spread of tetracycline-resistant strains in Madagascar. The high rate of resistance in Antananarivo, where the major Malagasy hospitals are located, could be due to easier access to drugs in the capital than in the other provinces.

Of the 60 randomly selected tetracycline-susceptible strains, 56 had the original antibiotype; four became susceptible to vibriostatic agent O129 and to all the antibiotics tested, except trimethoprim. Four (3.5%) of the 115 strains tested (55 tetracycline-resistant and 60 tetracycline-susceptible strains) on a large panel of antibiotics were susceptible to trimethoprim-sulfamethoxazole. As usually observed in other African cholera-endemic countries (*6*), only a small proportion of the strains were susceptible to trimethoprim-sulfamethoxazole, one of the most frequently dispensed drugs.

Faced with this first emergence of cholera in Madagascar and its rapid spread, medical authorities reacted immediately by using doxycycline as chemoprophylaxis (contrary to World Health Organization recommendations [*7*]), probably because of its easy availability**.**

Our study demonstrates that 2 years after the epidemic began, neither trimethoprim-sulfamethoxazole nor tetracycline, the two first-line drugs used in Madagascar, can be recommended any longer for treating severe cases of cholera. This may represent a critical public health problem in the country, especially as most of the population cannot afford more effective but expensive antibiotics.

Therefore, Malagasy medical authorities should a) abandon any systematic chemoprophylaxis, b) advise only oral rehydration therapy for mild-to-moderate cases, and c) reserve antibiotic therapy for severe illness (*7*). These measures against the cholera epidemic should be accompanied by general reinforcement of microbiologic surveillance to monitor antibiotic resistance so that the island can respond effectively to any future bacterial epidemics.
